# Anxiety and Alzheimer’s disease pathogenesis: focus on 5-HT and CRF systems in 3xTg-AD and TgF344-AD animal models

**DOI:** 10.3389/fnagi.2023.1251075

**Published:** 2023-11-10

**Authors:** Nicole C. Reyna, Benjamin J. Clark, Derek A. Hamilton, Nathan S. Pentkowski

**Affiliations:** Department of Psychology, University of New Mexico, Albuquerque, NM, United States

**Keywords:** Alzheimer’s disease, corticotropin releasing factor, serotonin, stress, HPA Axis

## Abstract

Dementia remains one of the leading causes of morbidity and mortality in older adults. Alzheimer’s disease (AD) is the most common type of dementia, affecting over 55 million people worldwide. AD is characterized by distinct neurobiological changes, including amyloid-beta protein deposits and tau neurofibrillary tangles, which cause cognitive decline and subsequent behavioral changes, such as distress, insomnia, depression, and anxiety. Recent literature suggests a strong connection between stress systems and AD progression. This presents a promising direction for future AD research. In this review, two systems involved in regulating stress and AD pathogenesis will be highlighted: serotonin (5-HT) and corticotropin releasing factor (CRF). Throughout the review, we summarize critical findings in the field while discussing common limitations with two animal models (3xTg-AD and TgF344-AD), novel pharmacotherapies, and potential early-intervention treatment options. We conclude by highlighting promising future pharmacotherapies and translational animal models of AD and anxiety.

## 1. Introduction

Currently over 55 million people worldwide are diagnosed with dementia, with 60–70% of dementia cases caused by Alzheimer’s disease (AD; World Health Organization, 2023). In the United States, over 6 million people are currently diagnosed with AD, with this number projected to rise due to the large aging population (Zhao, 2020; Alzheimer’s Association, 2023). There are distinct neurobiological changes that occur in AD patients, including deposition of amyloid-beta (Aβ) plaques, neurofibrillary tangles of tau, neuroinflammation, and neuronal loss (Duyckaerts et al., 2009; Zhao, 2020). This neuropathological burden of Aβ and tangles in the brain leads to neurodegeneration, with key areas such as the hippocampus, frontal cortex, and raphe nucleus exhibiting evidence of synaptic dysfunction, severe neuronal loss, and tissue atrophy (Goedert and Spillantini, 2006; Duyckaerts et al., 2009). Importantly, it has been reported that neurobiological changes begin years before initial AD diagnosis, even in the absence of cognitive changes or symptoms (Müller-Spahn, 2003; Sperling et al., 2011). Given the extent of irreversible neurobiological changes that occur in AD, it is critical that the field strive to promote preventative and early-stage AD treatments.

AD patients are diagnosed on a continuum consisting of Preclinical AD, Mild Cognitive Impairment (MCI), and Mild, Moderate or Severe Dementia due to AD (Sperling et al., 2011; Zhao, 2020; Alzheimer’s Association, 2023). There are several risk factors for developing AD, including age, genetics, and family history (Zhao, 2020; Alzheimer’s Association, 2023). Recent research has suggested a set of modifiable risk factors for developing AD, including smoking, cardiovascular health, low socioeconomic status (SES), fewer years of formal education, and poor sleep quality (Alzheimer’s Association, 2023). Among the modifiable risk factors, perhaps the most intriguing is oxidative and psychological stress. Research has shown that early AD pathogenesis may result in subtle neuropsychological changes, including increased anxiety, depression, or agitation (Schmid et al., 2013; Escher et al., 2019). The hypothalamic–pituitary–adrenal (HPA) axis is a critical system responsible for the body’s response to a perceived or actual threat (i.e., stressor), as well as the return to homeostasis following stress exposure. Homeostasis is achieved through neurological coordination between endocrine, autonomic, and behavioral systems (Aguilera, 2011). Specifically, the HPA axis is activated when there is a perceived stressor leading to an increase in the release of corticotropin-releasing factor (CRF) and subsequent cortisol (corticosterone in rodents) release (Cook, 2004). Excessive exposure to cortisol can result in psychiatric, reproductive, cardiovascular, immune, and metabolic disorders (Aguilera, 2011; Justice, 2018). Importantly, overexposure to cortisol can result in oxidative stress, which has been linked to onset of early-AD neurobiological changes such as mitochondrial dysfunction and synaptic loss (Spiers et al., 2015; Tchekalarova and Tzoneva, 2023). Further, AD patients with posttraumatic stress disorder (PTSD) or generalized anxiety disorder (GAD) are more likely to experience hastened progression and worsening of AD symptoms compared to non-anxious patients (Justice, 2018; Escher et al., 2019; Tchekalarova and Tzoneva, 2023). One meta-analysis reports that anxiety symptoms are associated with a 29% increase in the risk for dementia (Santabárbara et al., 2019, 2020), while a recent meta-analysis reports no significant association between anxiety and AD neuropathology (Demnitz-King et al., 2023). Additionally, it remains unclear if increased anxiety precedes AD, is a consequence of neurodegeneration caused by AD, or is some combination of both possibilities. One study reports that high cerebrospinal fluid (CSF) markers of hyperphosphorylated tau and total tau but low CSF markers of Aβ(42) were associated with neuropsychological burden in cognitively normal adults (Krell-Roesch et al., 2022). Another study reports that anxiety symptomatology predicts Aβ accumulation in non-AD, cognitively normal adults (Johansson et al., 2020). Thus, the involvement of the HPA axis, anxiety, and AD symptomatology warrants further investigation.

There are two systems of interest that impact the HPA axis and AD symptomatology and have potential protective factors against developing AD: the CRF and serotonergic (5-HT) systems (Hajszan et al., 2005; Canet et al., 2019). It is important to note that other systems, such as the noradrenergic system, contribute to stress and AD; however, for the purpose of this specific review this will not be covered (see Poe et al., 2020 for a review). Critically, transgenic (Tg) animals treated with CRF type 1 receptor (CRFR1) antagonists exhibit slowed progression of AD symptomatology. Additionally, the raphe nucleus, which is responsible for 5-HT production in the brain, has significant neuronal loss and dense collections of neurofibrillary tangles in AD patients (Duyckaerts et al., 2009; Zhang C. et al., 2016). Recent research continues to point to the impact of stress on cortisol in AD. Clinical epidemiological studies report that individuals who experience chronic stress see increased incidents of AD neuropathology later in life (Machado et al., 2014; Justice, 2018). Further, AD pathogenesis in early-onset AD animal models consistently report accelerated deposition of Aβ and worse cognitive performance compared to control animals (Dong et al., 2004; Kang et al., 2007; Srivareerat et al., 2009; Devi et al., 2010; Huang et al., 2023). Given the intertwined nature of AD, CRF, and 5-HT, it is likely that there is a treatment capable of targeting these key systems. Future cognitive and pharmacotherapies may aim to regulate the HPA axis to prevent and treat AD symptoms, including anxiety. Importantly, neurodegeneration due to AD is estimated to start 20–30 years before the first signs of clinical symptoms. Identifying patients diagnosed with anxiety disorders such as PTSD and GAD could lead to the implementation of early-intervention treatments for AD. In this review, treatments targeting the HPA axis and anxiety, specifically CRF and 5-HT, will be described, with AD animal models evaluated on translational ability.

## 2. Potential early intervention: targeting preclinical AD and the HPA axis

### 2.1. Prioritizing preclinical AD diagnosis and treatment

AD is a unique form of dementia due to the extended preclinical and prodromal phases of the disease. Typically, subclinical cognitive and neuropsychiatric symptoms are present two decades before a formal diagnosis (Masters et al., 2015). Currently, those who are diagnosed at the preclinical stage of AD have a confirmatory diagnosis of Aβ accumulation through positron emission tomography (PET) or CSF analysis (Dubois et al., 2014; Masters et al., 2015). It is possible that screening for neuropsychiatric symptoms may help improve rates of early diagnosis, especially because most AD patients present with anxiety and/or depression, and have trouble sleeping years prior to their AD diagnosis (Belleville et al., 2014; Burke et al., 2018; Eratne et al., 2018). This increased comprehensive screening is critical because people who receive an earlier diagnosis have improved outcomes and slowed progression of the disease (Rasmussen and Langerman, 2019). The current FDA-approved treatments available can only be prescribed, at the earliest, in the MCI stage (Zhao, 2020). FDA-approved drugs Aducanumab and Lecanemab are both Aβ monoclonal antibody medications that break down Aβ plaques in the brain; however, these medications have severe side effects, including brain hemorrhaging and swelling (Zhao, 2020; Alzheimer’s Association, 2023). Given the improved methodology for diagnosing AD patients in the preclinical stage and lack of early-intervention pharmacotherapies, there is a critical need for improved early-intervention treatments. Due to the increased rates of anxiety in patients in the prodromal and preclinical stages of AD, drugs targeting anxiety symptoms and circuitry, such as the HPA axis, present a potential path forward.

### 2.2. Anxiety, AD, and the HPA axis

Approximately 40 million adults in the U.S. live with a diagnosed anxiety disorder (Merikangas et al., 2010). Additionally, 40% of AD patients report experiencing anxiety symptoms (Mendez, 2021). Early-life adversity and exposure to stress correlates strongly with later diagnosis of major depression disorder or anxiety disorders (Liu, 2017). The modifiable risk factors of AD and early-life adversity overlap significantly, with low SES/economic hardship, poor sleep quality, and psychological stress present in both measures. Neurobiologically, early-life adversity is linked to increased CRF type-1 receptor (CRF_1_) expression in the hypothalamus, amygdala, and prefrontal cortex (Wang et al., 2022). Those exposed to early-life adversity showed smaller hippocampal volume and HPA axis hypoactivity (Dahmen et al., 2018), while other individuals who experienced early-life adversity demonstrated HPA axis hyperactivity (Farrell et al., 2018). Additionally, abnormal HPA axis activity has been observed in those diagnosed with GAD and AD, with some studies reporting hypoactivity/reduced cortisol levels and others reporting hyperactivity/increased cortisol levels (Du and Pang, 2015; Juruena et al., 2020). This supports a hypothesis posited by Miller et al. that there are both HPA axis responders and non-responders to psychosocial stressors (Miller et al., 2013). More commonly, AD patients present with increased cortisol levels measured through saliva or plasma (Csernansky et al., 2006; Bangasser et al., 2017). CRF pathway activation is critical to increased cortisol levels due to its signaling and activation of the downstream G_s_, cAMP, PKA pathway, ultimately leading to pro-AD signaling and resulting in a hastened progression of the disease (Yan et al., 2018). This CRF system signaling was first characterized extensively by Vale et al., who described the ability of high potency CRF_1_ to stimulate adrenocorticotropic hormone and glucocorticoid secretion through the paraventricular nucleus of the hypothalamus (Vale et al., 1981). SSRIs affect the same pathway through upregulation of 5-HT_1A_ and subsequent inhibition of the G_s_, cAMP, PKA pathway. An example of this is the SSRI escitalopram, which mediates HPA axis activity via CRF inhibition (Tafet and Nemeroff, 2020). Interestingly, clinical populations show a reduction of the 5-HT_1A_ autoreceptor in the raphe nucleus after SSRI treatment (Gray et al., 2013). This finding has been consistent in the literature, with other studies reporting 5-HT_1A_ autoreceptors having increased desensitization compared to 5-HT_1A_ heteroreceptors. This is important to highlight when considering treatment with SSRIs for AD, as 5-HT_1A_ receptors are critical to hippocampal signaling, activation of growth factor-regulated signaling pathways, and neuronal myelination processes (Gray et al., 2013; Kroeze et al., 2015; Albert and Vahid-Ansari, 2019). Given the extensive involvement of both the CRF and 5-HT systems in HPA axis regulation and downstream AD pathogenesis, early-intervention treatments should consider prioritizing these two systems.

Clinical AD populations treated with SSRIs report enhanced memory and cognition (Mowla et al., 2007; Bartels et al., 2018; Elsworthy and Aldred, 2019; Lenze et al., 2023). Critically, AD patients with behavioral and cognitive deficits who receive treatment with SSRIs show significant improvements and hippocampal neurogenesis (Santarelli et al., 2003; Correia et al., 2021). However, the rodent models of AD yield mixed results. A more recent study found no differences in amyloid burden or cognitive performance in AD patients treated with an SSRI and cognitively normal controls (Bouter and Bouter, 2022). Further, one meta-analysis reported no significant differences between AD patients treated with SSRIs and AD patients not treated with SSRIs (Jones et al., 2016), while another meta-analysis reported an increased risk of dementia in people prescribed SSRIs, monoamine oxidase inhibitors, and tricyclics (Caballero et al., 2006; Popovic et al., 2015; Wang et al., 2018). Ultimately, there are few randomized control trials examining the effects of SSRIs on dementia patients. Despite the mixed findings in clinical populations, preclinical data suggest that SSRIs may reduce AD pathogenesis while improving behavioral and cognitive outcomes (Elsworthy and Aldred, 2019; Lenze et al., 2023). It is possible that a more targeted pharmacotherapy, such as a CRF_1_ antagonist, with or without a standard antidepressant, may produce more robust effects in clinical populations due to their potency and specificity compared to antidepressants.

## 3. Animal models of AD

### 3.1. Basic characteristics of Tg animal models

**Figure 1:**
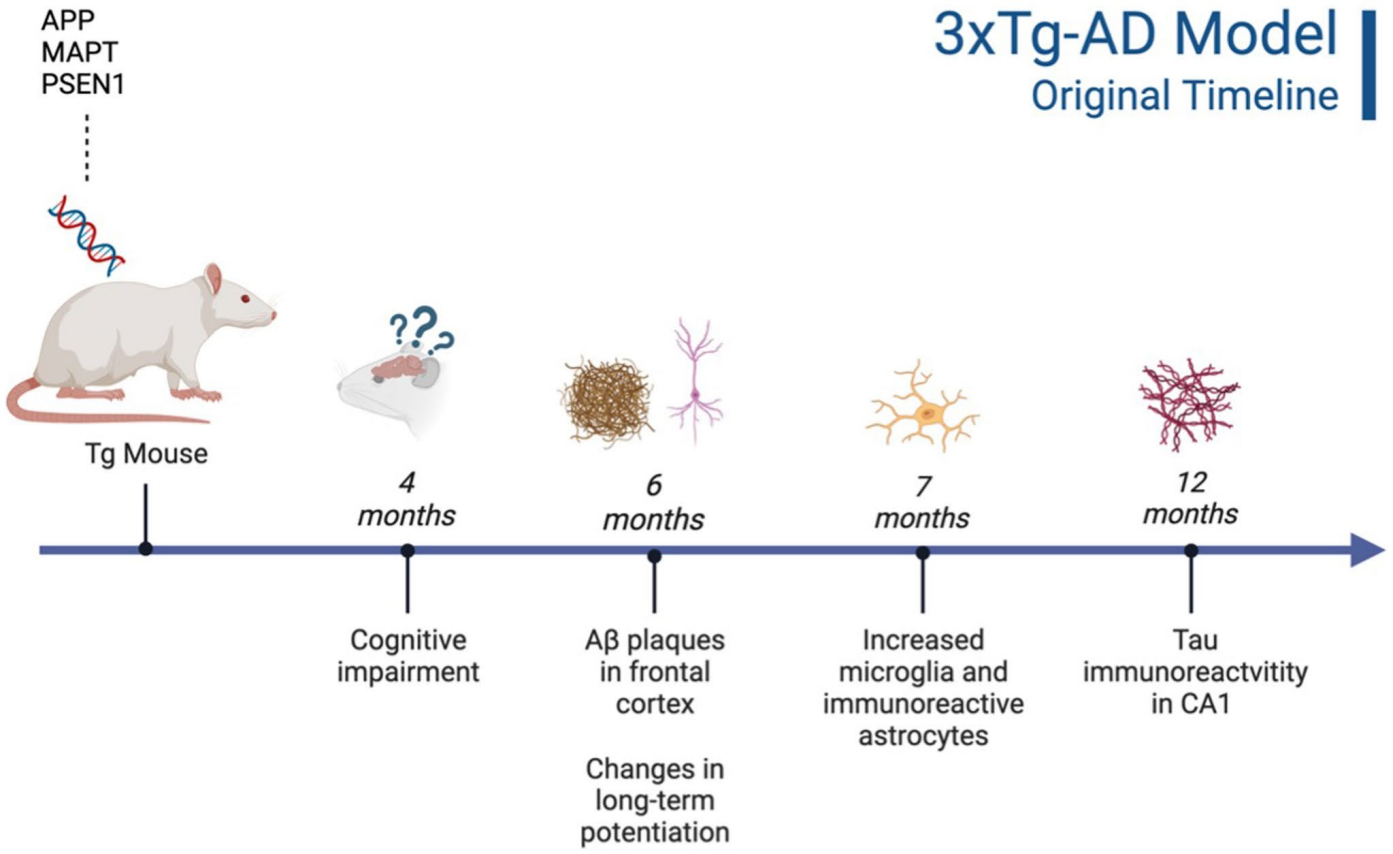
Timeline of AD pathogenesis in 3xTg-AD model (original characterization; Oddo et al., 2003). Created with BioRender.com.

**Figure 2:**
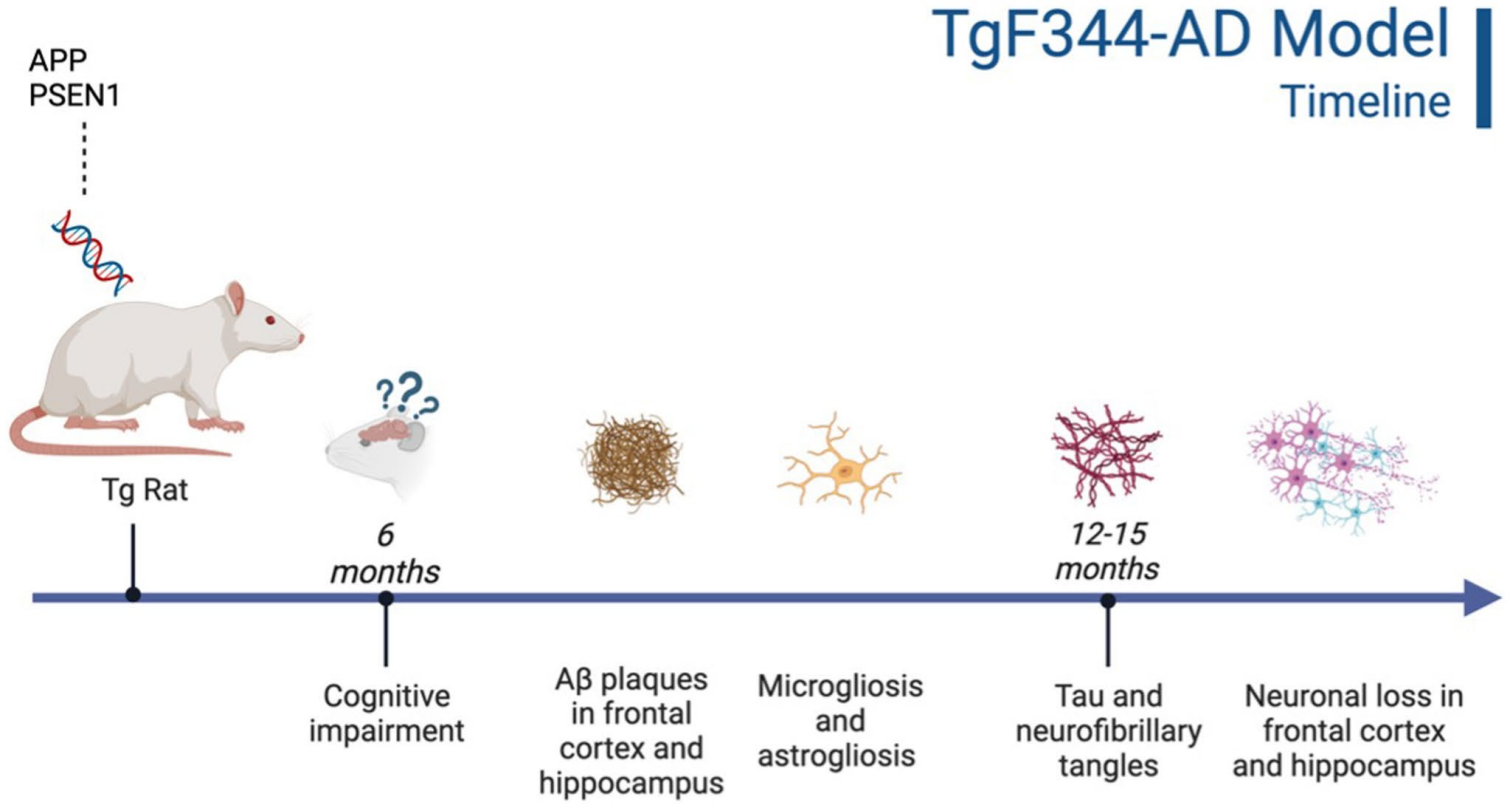
Timeline of AD pathogenesis in the TgF344-AD model (Cohen et al., 2013). Created with BioRender.com.

Currently, there are 214 animal models of early-onset AD (EOAD) available, with 197 AD mouse models and 17 AD rat models (Götz et al., 2018). Mouse models of AD are generally preferred for Tg experiments due to low breeding and husbandry costs and ease of gene manipulation. In contrast, rat models are generally preferred for behavioral tasks due to their increased sociality and increased translational relevance (Bryda, 2013). Regardless of species, there are five genes commonly targeted in AD Tg models: amyloid-precursor protein (APP), presenilin 1 (PS1), presenilin 2 (PS2), microtubule-associated protein tau (MAPT), and apolipoprotein E (APOE; Remy et al., 2014; Alzheimer’s Association, 2023). The APP, PS1, and PS2 genes are implicated in EOAD, while the APOE gene is implicated in late-onset AD (LOAD). MAPT is typically integrated into animal models that do not express robust tauopathy with other AD genetic manipulations alone. This could be a potential benefit of rat models, due to their natural expression of tau isoforms compared to mouse models that rely on integration of human mutant MAPT genes. Genes of animals can be manipulated in two ways, either through Tg technology or genome editing recombination. In contrast, genome editing recombination, such as homology directed recombination, knocks out or replaces the targeted AD genes, guaranteeing AD pathology expression in the selected rodents (Goedert and Spillantini, 2006; Filali et al., 2012; Götz et al., 2018; Alzheimer’s Association, 2023). Due to the abundance of AD animal models available, two AD models will be prioritized in the current review: the 3xTg-AD mouse model (see Figure 1) and the TgF344-AD rat model (see Figure 2). These models were chosen due to their common use in the literature and robust AD neuropathology expressed, including AB plaques and tau tangles.

**Figure 3:**
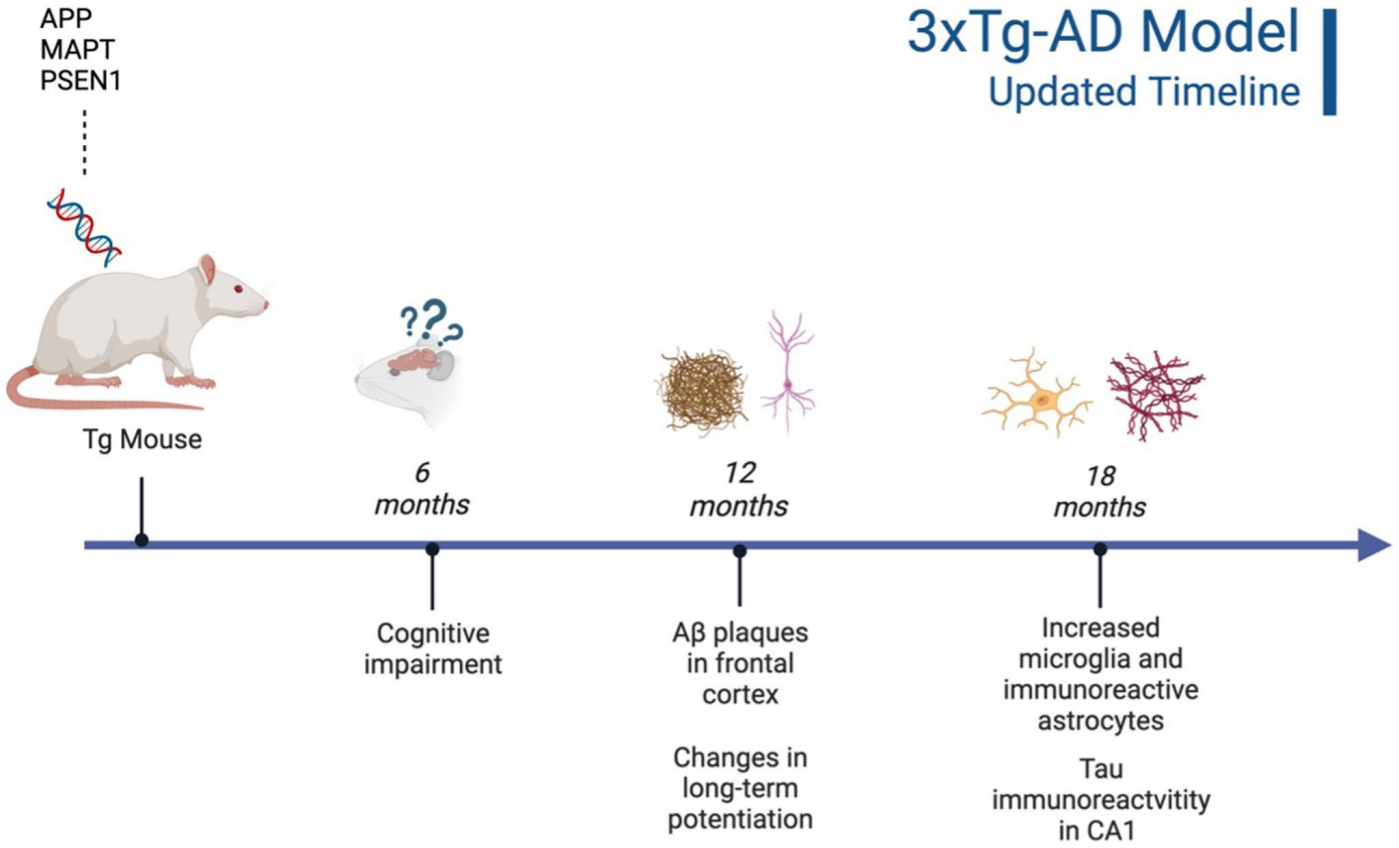
Updated characterization of AD pathogenesis in the 3xTg-AD model (Javonillo et al., 2022). Created with BioRender.com.

It is important to highlight the potential differences in AD strains since their original development. Due to TgF344-AD rodents being developed and characterized more recently, there is less concern regarding genetic drift and consistency between Tg colonies, with multiple papers replicating the original phenotype findings reported by Cohen et al. (2013), Anckaerts et al. (2019), and Fowler et al. (2022). However, 3xTg mice were developed 20 years ago (Oddo et al., 2003) and there is evidence of genetic drift impacting both behavioral and neural AD pathology. A comparative study looking at the drift was conducted by the LaFerla Lab, which originally developed the 3xTg-AD model. The authors report that compared to their original study in 2003, 3xTg-AD mice develop age-dependent Aβ and tangles, but this development occurs significantly later in the model with AD pathology previously present at 12 mo. and now present at 18 mo. (Javonillo et al., 2022). According to several studies, Aβ plaque development occurs in female 3xTg-AD mice from 12 to 18 months, with males showing significantly less Aβ and hyperphosphorylated tau deposition at 18 months (Carroll and Pike, 2008; Creighton et al., 2019; Javonillo et al., 2022). Additionally, female 18-month-old 3xTg-AD mice present with increased microglial density compared to males (Javonillo et al., 2022; see Figure 3). Although genetic drift is present in the 3xTg mouse model, it is important to consider that there is no ideal animal model for EOAD. Scientists should remain vigilant for potential genetic drift and subsequent behavioral changes that may occur. In-depth phenotype characterization is necessary for each model in order to properly assess and quantify AD pathological and behavioral changes in Tg models (Javonillo et al., 2022). The present review aims to investigate two EOAD animal models that expressed robust AD pathology while prioritizing research that manipulated or analyzed the serotonergic and CRF systems. Given these parameters, the models most frequently observed in the literature were the 3xTg-AD mouse and TgF344-AD rat models. However, it is important to note that the effects of stress, anxiety, cortisol, and AD pathogenesis should be investigated in all AD Tg models currently available.

### 3.2. 3xTg-AD mouse model: neurobiology and behavioral observations

**Figure 4:**
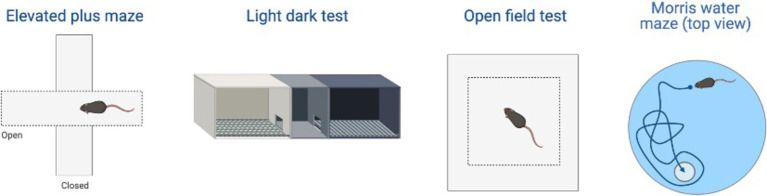
Examples of the behavioral test examined in the review including: elevated plus maze (EPM), light-dark test (LDT), open field test (OFT), and Morris water task (MWT). Created with BioRender.com.

This triple Tg model expresses the APP, PS1, and MAPT genes with Aβ plaques present at 6 months and tau/tangles present at 12 months. Additionally, 3xTg mice show synaptic dysfunction, including long-term potentiation deficits (Oddo et al., 2003; Blázquez et al., 2014). Considering the genetic drift of the 3xTg-AD mice, deposition of Aβ and tau, as well as LTP deficits, occur around 18 months, with more pronounced deposition reported in female 3xTg-AD mice (Javonillo et al., 2022). Increased anxiety, defined as the behavioral and/or physiological response to perceived threat, is typically measured in animal models through testing in elevated plus-maze (EPM) or the light–dark test (LDT; for a review of animal models see Pentkowski et al., 2021). In the EPM, rodents classified as exhibiting enhanced anxiety-like behavior spend more time in the closed arms compared to the open arms. Similarly, for the LDT, rodents exhibiting heightened anxiety-like behavior tend to spend more time in the dark compartment compared to the light compartment. Both tests capitalize on rodents’ tendency to seek shelter from potential predators by staying in dark, enclosed spaces. Further, entries into the light, exposed environments are recorded to assess for exploratory behavior (see Figure 4). Rodents that explore more are presumed to be less anxious (McCormick and Green, 2013). Extremely conflicting results have been reported in anxiety measures in the 3xTg-AD model. Prior to AD pathology being expressed, 3xTg-AD mice at 3 and 4 months do not exhibit enhanced anxiety-like behavior on the EPM, with Tg female mice spending increased time in the open arms compared to wild-type mice (WT; Pairojana et al., 2021; Várkonyi et al., 2022). Anxiety-like behaviors in LDT continue to be inconsistent at this age, with 4-month-old WT mice spending more time in the dark compartment compared to Tg mice (Várkonyi et al., 2022). At 6 months, 3xTg-AD behavior in the EPM is varied, with one study reporting that Tg mice spent more time in the closed arms and made fewer entries into the open arms (Zhang Y. L. et al., 2016), while other studies observed no differences between Tg and WT mice (Hebda-Bauer et al., 2013; Pairojana et al., 2021). For the LDT, 6-month-old 3xTg-AD mice spent more time in the dark compartment, consistent with elevated anxiety-like behavior (Zhang C. et al., 2016; refer to Table 1). Older 3x-Tg mice (7.5–12 months old) did not differ in amount of time spent in the open arms of the EPM or the amount of locomotor activity exhibited compared to controls. This observation suggests that the mice are not exhibiting enhanced anxiety at this time point, despite the progression of positive lesions and cognitive deficits (Sterniczuk et al., 2010; Pairojana et al., 2021). In the LDT, 8–12-month-old 3xTg mice spent significantly more time in the dark compartment and exhibited decreased locomotion, which is consistent with increased anxiety-like behavior (Blázquez et al., 2014; Várkonyi et al., 2022; refer to Table 2).

**Table 1:** Summary of LDT effects in 3xTg-AD mouse model.

Difference between WT and Tg	Age (months)	Sex	Behavior	Effect sizes/significance (*η*^2^, *d*, *p*)	Authors
✓	4, 8	M	WT spent more time in the dark compartment compared to Tg.	*η*^2^ = 0.22 (Time in compartment)	Várkonyi et al. (2022)
			Motivation to explore decreased significantly in 8 mo. Tg animals.	*η*^2^ = 0.09 (Motivation)	
✓	6	M, F	White box entries and time spent in light significantly decreased for Tg	*p* < 0.05	Zhang Y. L. et al. (2016)
				*p* < 0.05	
✓	12	M, F	Tg mice spent less time on the white side; this effect is more pronounced in Tg M.	*η*^2^ = 0.12 (Tg, M and F)	Blázquez et al. (2014)
				*η*^2^ = 0.09 (Tg, M)	

**Table 2:** Summary of EPM effects in 3xTg-AD mouse model.

Difference between WT and Tg	Age (months)	Sex	Behavior	Effect sizes/significance (*η*^2^, *d*, *p*)	Authors
✘	3, 9, 12	M	No differences between WT and Tg	NR	Pairojana et al. (2021)
✓	3, 6, 9	F	Tg spent more time in the open arms and less time in the closed arms.	*η*^2^ = 0.46 (3mo. Open arm)	Pairojana et al. (2021)
				*η*^2^ = 0.42 (3mo. Closed arm)	
				*η*^2^ = 0.26 (6 mo. Open arm)	
				*η*^2^ = 0.30 (9 mo. Open arm)	
✘	4, 8	M	No differences between WT and Tg in open arm time or locomotion	*η*^2^ = 0.01 (Open arm time)	Várkonyi et al. (2022)
				*η^2^ = 0.07 (Locomotion)	
✘	6	M, F	No differences between WT and Tg in distance traveled or locomotor activity	*η*^2^ = 0 (Distance traveled)	Hebda-Bauer et al. (2013)
				*η*^2^ = 0 (Locomotion)	
✓	6	M	Tg spent decreased time in the closed arms	*d* = 2.99	Pairojana et al. (2021)
✓	6	M, F	Tg animals had more entries in the closed arms. Tg animals spent less time in the open arms.	*p* = 0.006 (Closed arm entries)	Zhang C. et al. (2016)
				*p* = 0.004 (Open arm time)	
✘	7.5–11	F	No significant differences between WT and Tg	*η*^2^ = 0.03	Sterniczuk et al. (2010)
✘	12	F	No differences between WT and Tg	NR	Pairojana et al. (2021)

Other behaviors measured in AD animal models include deficits in locomotion, and spatial learning and navigation. The Morris Water Task (MWT) measures spatial learning ability by training rodents to swim to a platform over a period of days, while the open-field task (OFT) measures locomotor activity and exploratory behavior organized around salient locations termed a “home base” (Whishaw and Tomie, 1996; Seibenhener and Wooten, 2015; Thompson et al., 2018; Donaldson et al., 2019; see Figure 2). Importantly, the OFT can also be used to assess anxiety-like behaviors due to thigmotaxic behaviors present in animals and the proclivity of animals to spend long periods of time at home base locations (Whishaw et al., 2006). Due to the increase in cognitive and neurological deficits as AD progresses, it should be expected that mice perform progressively worse on the MWT and have a reduction in locomotion. Few studies observed 3xTg-AD mice prior to development of AD pathology; however, one study reports that 4-month-old 3xTg-AD mice show no deficits in spatial learning compared to WT mice (Várkonyi et al., 2022). Further, 3–4-month-old 3xTg-AD mice exhibit decreased locomotion and increased grooming behaviors compared to WT controls (Pairojana et al., 2021; Várkonyi et al., 2022). At the onset of AD pathogenesis, 8-month-old 3xTg mice spent more time locating the platform in the MWT on the final of training compared to their WT counterparts (Várkonyi et al., 2022; refer to Table 3). 6–8-month-old 3xTg mice have inconsistent locomotor behaviors in the OFT, with some mice exhibiting less active movement and others not differing from WT controls (Sterniczuk et al., 2010; Hebda-Bauer et al., 2013; Zhang Y. L. et al., 2016; Pairojana et al., 2021; Várkonyi et al., 2022). Older 3xTg-AD mice (12–15 months old) perform consistently worse on the MWT compared to WT controls, showing severe deficits in spatial memory. However, it is important to note that these effects were captured on the first days of training. The differences between Tg and WT 3xTg-AD mice were not seen on the final days of MWT testing when performance was at asymptote (Blázquez et al., 2014; Torres-Lista et al., 2015). Most studies report a significant reduction in locomotion in the OFT in older 3xTg mice, but one study found no differences in locomotor behavior compared to WT controls (Sterniczuk et al., 2010; Blázquez et al., 2014; Torres-Lista et al., 2015; Pairojana et al., 2021). Overall, behavior measured in the MWT and OFT varied between labs and studies (refer to Table 4).

**Table 3:** Summary of MWT in 3xTg-AD mouse model.

Difference between WT and Tg	Age (months)	Sex	Behavior	Effect sizes/significance (*η*^2^, *d*, *p*)	Authors
✘	4	M	No significant differences between WT and Tg at 4 mo.	*p* > 0.05 (4 mo.)	Várkonyi et al. (2022)
✓	8		Tg found platform slower than WT on day 5.	*η*^2^ = 0.57 (8 mo.)	
✓	12, 15	M, F	Tg have worse spatial learning acquisition (on day 1 and 2).	*η*^2^ = 0.17 (12 mo. M)	Blázquez et al. (2014)
				*η*^2^ = 0.32 (12 mo. F)	
				*η*^2^ = 0.23 (15 mo. M)	
				*η*^2^ = 0.29 (15 mo. F)	
✓	13	M	Tg had worse spatial learning acquisition on day 1.	*η*^2^ = 0.05	Torres-Lista et al. (2015)

**Table 4:** Summary of OFT effects in 3xTg-AD mouse model.

Difference between WT and Tg	Age (months)	Sex	Behavior	Effect sizes/significance (*η*^2^, *d*, *p*)	Authors
✓	3, 12	M, F	Tg had significantly decreased locomotion.	*η*^2^ = 0.31 (3 mo.)	Pairojana et al. (2021)
✘	6		No significant differences in locomotion.	*η*^2^ = 0.55 (12 mo.)	
✓	9		M Tg had significantly decreased speed and locomotion compared to Tg F and WT.	NR (6 mo.)	
				*d* = 6.67 (9 mo., Speed)	
				*d* = 14.89 (9 mo., Locomotion)	
✓	4, 8	M	Tg spent less time in active movement.	*η*^2^ = 0.36 (4 mo.)	Várkonyi et al. (2022)
				*η*^2^ = 0.29 (8 mo.)	
✘	6		No difference in total distance traveled between Tg and WT.	*p* > 0.05 (Distance)	Zhang C. et al. (2016)
✓			WT spent more time in center.	*p* < 0.05 (Center)	
✘	6	M, F	No differences in locomotor activity between Tg and WT.	*η*^2^ = 0.04 (Locomotion)	Hebda-Bauer et al. (2013)
✓			Tg spent more time in the center.	*η*^2^ = 0.21 (Center)	
✘	7.5–11	F	No significant differences in locomotion between Tg and WT.	*η^2^ = 0.39 (Distance)	Sterniczuk et al. (2010)
✓			Tg spent more time in the center compared to WT.	*η^2^ = 0.50 (Speed)	
				*η*^2^ = 0.11 (Center)	
✓	12, 15	M, F	Tg exhibit less exploratory behavior (12 mo.).	*η*^2^ = 0.19 (12 mo.)	Blázquez et al. (2014)
			Tg mice exhibit more freezing behavior (15 mo.).	*η*^2^ = 0.24 (15 mo.)	
✓	12	M	Grooming behaviors appeared later in the task.	*d* = 1.38	Torres-Lista et al. (2015)

Although neurobiological changes reported in the 3xTg-AD mouse model remain consistent with development of Aβ plaques (6 months/18 months) and tau/tangles (12 months/18 months; see Figures 1 and 3), behavioral results are more inconsistent (Oddo et al., 2003; Zhang C. et al., 2016). For the anxiety measures using the EPM and LDT, LDT findings were more consistent, with 3xTg-AD mice consistently exhibiting elevated anxiety-like behavior, with increased time spent in the dark compartment and fewer entries into the light compartment compared to controls. The EPM findings varied heavily, with some Tg mice spending increased time in the open arms and others more time in the closed arms. In the MWT, 3xTg-AD mice generally performed worse than their WT counterparts; however, OFT findings were more varied. At all ages, 3xTg-AD mice expressed differing behavior, with some Tg mice expressing decreased locomotion and speed while other Tg mice did not differ significantly from WT controls. Although the behavioral results were more varied, the 3xTg-AD model is still an ideal model for examining anxiety-like behaviors and the impact on AD. This is due to the robust AD pathology expressed, as well as enhanced anxiety-like behavior and locomotor deficits present from 6–12 months of age. Despite genetic drift reported in the 3xTg-AD model, there is still consistent behavioral and AD pathology reports at 18 months. The 3xTg-AD model is heavily cited and widely used, allowing for robust meta-analyses and comparisons of colonies. Additionally, the 3xTg-AD model may be more translational to a clinical population compared to other mouse AD models due to the gradual onset of AD symptoms and pathology (Liu et al., 2021). However, the implantation of the tau gene, MAPT, may cause this model to be slightly less translationally valid compared to AD models that do not rely on genetic manipulation for expression of tau. Future studies should verify the presence of microglia, Aβ, and tau, to ensure standardization across various colonies, labs, and studies.

### 3.3. TgF344-AD rat model: neurobiology and behavioral observations

This Tg rat model expresses both the mutant human amyloid precursor protein (APP_swe_) and presenilin 1 (PS1ΔE9) genes. The model consistently shows age-dependent cerebral Aβ plaque and tau deposition, neuronal loss, and cognitive disturbance (Cohen et al., 2013). Typically, TgF344-AD rats begin to express Aβ plaque deposition at 5–6 months, prior to marked cognitive deficits being observed. This is followed by robust tauopathy at 12–16 months and neuronal loss and neuroinflammation present around 16 months (Cohen et al., 2013; Wu et al., 2020) For rat studies, primarily the EPM and OFT were used to measure anxiety-like behavior, with measures examining exploratory behavior via time spent and entries into the open arms (EPM), and time spent in the center of the area (OFT; see Figure 4). Younger TgF344-AD rats yielded some mixed behavioral results. 2- and 6-month-old TgF344-AD rats did not exhibit altered anxiety-like behavior in the EPM or OFT according to some studies (Wu et al., 2020; Kelberman et al., 2022), while other studies reported increased anxiety-like behavior demonstrated by reduced open arm time in the EPM and reduced locomotion in the OFT in TgF344-AD rats aged 4.5–6.5 months (Pentkowski et al., 2018, 2022; Saré et al., 2020). Most of the variability in the anxiety behavioral measures occurs in TgF344-AD rats aged 6 months, with studies finding no differences between TgF344-AD and WT in the OFT, with TgF344-AD rats spending less time in the center of the OFT compared to WT rats, or TgF344-AD hypoactivity (Cohen et al., 2013; Saré et al., 2020; Kelberman et al., 2022). At 9 months, findings are more consistent, with two studies finding increased anxiety-like behaviors in Tg rats. Specifically, compared to WT rats, Tg rats spent less time in the open arms of the EPM and less time in the center of the open field (Tournier et al., 2021; Srivastava et al., 2023). After rats were aged 12 months or older, anxiety-like behavior was more consistently demonstrated in both the EPM and OFT (Cohen et al., 2013; Saré et al., 2020; Wu et al., 2020; Kelberman et al., 2023). However, some studies still report no differences between older TgF344-AD and WT rats during testing in the EPM and OFT (Saré et al., 2020; Srivastava et al., 2023; refer to Tables 5, 6).

**Table 5:** Summary of EPM effects in TgF344-AD rat model.

Difference between WT and Tg	Age (months)	Sex	Behavior	Effect sizes/significance (*η*^2^, *d*, *p*)	Authors
✘	2, 12	M	No differences between Tg and WT (2 mo.).	NR (2 mo.)	Wu et al. (2020)
✓			Tg spent less time in the open arms (12 mo.).	*p* = 0.001 (12 mo.)	
✓	4.5–6.5	M, F	Tg spent less time in the open arms.	*η*^2^ = 0.37	Pentkowski et al. (2018)
✘	6, 12	M, F	No differences between Tg and WT (6 mo.).	*η*^2^ = 0.00 (6 mo.)	Kelberman et al. (2022)
✘			No differences between Tg and WT (12 mo.).	*η*^2^ = 0.00 (12 mo.)	
✓	6–7	M	Tg spent less time in the open arms.	*η*^2^ = 0.29	Pentkowski et al. (2022)
✓	9	M, F	Tg spent less time in the open arms.	*d* = 1.21	Tournier et al. (2021)
✘	9, 12	M, F	No differences between Tg and WT (9 mo.)	NR (9 mo.)	Srivastava et al. (2023)
✘			No differences between Tg and WT (12 mo.).	NR (12 mo.)	

**Table 6:** Summary of OFT effects in TgF344-AD rat model.

Difference between WT and Tg	Age (months)	Sex	Behavior	Effect sizes/significance (*η*^2^, d, *p*)	Authors
✘	2, 12	M	No differences between Tg and WT (2 mo.)	NR (2 mo.)	Wu et al. (2020)
✓			Tg rats spent less time in the center (12 mo.)	*p* < 0.01 (12 mo.)	
✓	6, 12	M, F	12 mo. Tg animals moved less compared to 6 mo. Tg animals.	*η*^2^ = 0.17 (Locomotion)	Kelberman et al. (2022)
✓			Tg animals spent less time in the center (6 mo., 12 mo.)	*η*^2^ = 0.06 (Center Time)	
✘	6, 15	M, F	No difference between Tg and WT (6 mo.)	*p* > 0.05 (6 mo.)	Cohen et al. (2013)
✓			Tg reared significantly more. (15 mo.)	*p* < 0.05 (15 mo.)	
✘	6, 12, 18	M, F	No differences between Tg and WT (M).	*η*^2^ < 0.03 (M, 6, 12, 18 mo.)	Saré et al. (2020)
✓			Female Tg hypoactivity (6 and 12 mo.)	*η*^2^ < 0.03 (F, 6, 12 mo.)	
✘			No significant differences between Tg and WT females (18 mo.)		
✓	9, 12	M, F	Tg F spent less time in center compared to M Tg and WT (9 mo.).	*p* < 0.05 (9 mo.)	Srivastava et al. (2023)
✘			No significant differences between Tg and WT (12 mo.).	NR (12 mo.)	

To assess cognitive deficits in spatial learning and navigation, studies utilized the MWT (see Figure 4). One study examined cognition and spatial learning in TgF344-AD rats prior to AD pathogenesis and found no significant differences in spatial learning at 4.5–6.5 months (Pentkowski et al., 2018). After 6 months, TgF344-AD rats show deficits in initial acquisition and take longer paths to the platform (Bernaud et al., 2022). However, another study reported no differences in path length between Tg and WT rats at 7–8 months (Berkowitz et al., 2018). Studies continue to report Tg rats taking longer paths at 9–10 months (Berkowitz et al., 2018; Bernaud et al., 2022), yet one study reports no differences in locating the platform during the probe trial in 9-month-old Tg and WT animals (Srivastava et al., 2023). TgF344-AD rats aged 12 months or older continue to execute longer paths and less direct trajectories and have more trouble locating the platform (Bernaud et al., 2022; Bac et al., 2023; Srivastava et al., 2023), with only one study reporting no differences between TgF344-AD and WT rats at 12 months (Kelberman et al., 2023; refer to Table 7).

**Table 7:** Summary of MWT effects in TgF344-AD rat model.

Difference between WT and Tg	Age (months)	Sex	Behavior	Effect sizes/significance (*η*^2^, d, *p*)	Authors
✘	4.5–6.5	M, F	No differences between Tg and WT on probe trial.	*η*^2^ = 0.06 (Platform preference)	Pentkowski et al. (2018)
				*η*^2^ = 0.02 (Proximity)	
✓	4–5, 7–8, 10–11	M, F	Tg had longer paths to the platform (10–11 mo.).	*η*^2^ = 0.18 (10–11 mo.)	Berkowitz et al. (2018)
✘			No significant differences in path length between Tg and WT (4–5 mo. and 7–8 mo.)	NR (4–5–7–8 mo.)	
✘	6–8, 18–21	M, F	No differences in locating the platform (6–8 mo.).	*η*^2^ = 0.06 (6–8 mo.)	Bac et al. (2023)
✓			Tg rats with high LI scores had longer paths (18–21 mo.)	NR (18–21 mo.)	
✓	6, 9, 12	F	Tg rats swam longer paths and took longer locate platform (6 mo., 9 mo., 12 mo.).	*η*^2^ = 0.30 (6 mo.)	Bernaud et al. (2022)
✓				*η*^2^ = 0.37 (9 mo.)	
✓				*η*^2^ = 0.29 (12 mo.)	
✘	9, 12	M, F	No significant differences between Tg and WT in probe trial (9 mo.).	NR (9 mo.)	Srivastava et al. (2023)
✓			Tg spent less time in platform area during probe trial. (12 mo.)	*p* < 0.01 (12 mo.)	
✘	12	M, F	No differences between Tg and WT on probe trial (12 mo.).	*η*^2^ = 0.07 (12 mo.)	Kelberman et al. (2022)

Neurobiological changes in TgF344-AD rats remain consistent throughout studies, with this model expressing robust AD neuropathology not expressed in many other animal models of AD (Saré et al., 2020; Wu et al., 2020; See Figure 2). A distinct difference between the TgF344-AD and the 3xTg-AD models is the expression of neuronal loss, gliosis, neuroinflammation, and tau expression in TgF344-AD rats that is not reliant on implanted mutated human tau. Given this robust and complete AD pathology expression, this rat model may generate more translational results than mouse models, including the 3xTg-AD model. Further, the TgF344-AD rat model yielded more consistent cognitive and behavioral results, with TgF344-AD rats aged more than 6 months typically exhibiting more anxiety-like behavior and deficits in spatial navigation and learning. However, this model was developed a decade after the 3xTg-AD model and needs more extensive research into the consistency of behavioral and cognitive results reported in the present review (Oddo et al., 2003; Cohen et al., 2013).

## 4. Novel pharmacotherapies: CRF antagonists and SSRIs in AD

### 4.1. CRF in AD models

Dysregulation of the HPA axis and CRF_1_ are critical to AD pathogenesis, and investigation of these systems could lead to novel treatments for both EOAD and LOAD due to the modifiable environmental risk factors implicated in the onset of both EOAD and LOAD (Cañete et al., 2015). Indeed, chronic stressor-induced HPA axis dysregulation leads to increased activation, synthesis, and release of glucocorticoids, which can have deleterious consequences on brain morphology and function (De Bellis et al., 1999), as well as the diminished ability to suppress cortisol secretion, which is seen in AD patients and rodents in the preclinical/prodromal stage (Csernansky et al., 2006; Rothman et al., 2012; Nguyen et al., 2020). Critical to HPA axis regulation are glucocorticoid receptors (GR) and CRF_1_. In rodents, often females have more central CRF_1_ expression compared to males (Donner and Lowry, 2013; Bangasser et al., 2017), which may represent one mechanism underlying higher rates of AD in females. Further, in AD rodent models, increased cortisol levels are thought to result from increased GR and CRF_1_ expression in key areas such as the hippocampus and cortex (Dong et al., 2008; Rissman et al., 2012; see Mohammadi et al., 2022 for a review). The impacts of HPA dysfunction, GR and CRF_1_ expression, and novel pharmacotherapies targeting these systems will be presented below.

### 4.2. CRF trends and treatments in rodent AD models

Generally, 3xTg mice aged 3–4 months do not exhibit differing corticosterone (CORT) levels compared to controls; however, some 3xTg males did exhibit increased GR mRNA expression in the hippocampus and paraventricular nucleus, suggesting a possible progression toward increased CORT in this AD model (Hebda-Bauer et al., 2013; Nguyen et al., 2020). In the early stages of AD progression, CORT measurements vary. Additionally, dexamethasone non-suppression was found in 4-month-old 3xTg mice (Várkonyi et al., 2022), suggesting reduced levels of GR. Higher CORT levels were reported in 6-month-old 3xTg-AD males and females, while another study reported no differences between 3xTg-AD and WT mice (Hebda-Bauer et al., 2013; Baeta-Corral et al., 2023). At 9 months, 3xTg-AD males have higher basal CORT levels compared to 3xTg-AD females and WT (Clinton et al., 2007; Nguyen et al., 2020). 3xTg-AD animals aged beyond 12 months consistently have increased CORT levels compared to controls, which aligns with full expression of AD pathology in this model (Rothman et al., 2012; Muntsant and Giménez-Llort, 2021). Overall, this remains consistent with the findings of age-dependent increases in cortisol serum levels (Green et al., 2006). This steady increase in GR and subsequent CORT measurements suggests that the HPA axis is severely dysregulated in the 3xTg-AD model. Thus, novel pharmacotherapies could target GR and CRF_1_ receptors to reduce downstream G_s_, cAMP, PKA activation and increased cortisol release. Indeed, chronic administration of the GR antagonist (RU486) in 10-month-old mice reversed Aβ deposition and reduced tau/NFT (Baglietto-Vargas et al., 2013). This trend is well supported in similar mouse models of AD, with Tg mice treated with CRF_1_ antagonists (Antalarmin, R121919) demonstrating lower levels of Aβ, tau, and CORT (Dong et al., 2014; Campbell et al., 2015; Zhang Y. L. et al., 2016; Zhang and Rissman, 2017). Research utilizing AD rat models is more limited because AD rat models were developed more recently. However, some data from studies indicate increased CORT levels when Aβ is present (Brureau et al., 2013; refer to Table 8). Additionally, current trends in the literature suggest that due to the well-established behavioral phenotype of TgF344-AD rats, future research should implement novel pharmacotherapies aimed at HPA axis regulation (Justice, 2018; Pentkowski et al., 2022).

**Table 8:** Summary of CRF trends in AD models.

Difference between WT and Tg	Age (months)	Model	Sex	CRF changes observed	Effect sizes/significance (*η*^2^, *d*, *p*)	Authors
✓	1.5–2	Sprague–Dawley (injected with Aβ_25-35_)	M	Tg Plasma CORT levels were higher compared to WT (6 weeks post injection).	*p* < 0.38	Brureau et al. (2013)
✓	3–4	3xTg-AD	M, F	M Tg rats have increased GR mRNA levels compared to M WT	*η*^2^ = 0.02	Hebda-Bauer et al. (2013)
✓				M WT have increased CRH mRNA in the PVN compared to Tg M.	*η*^2^ = 0.02	
✓				F Tg have increased GR mRNA levels in the CeA compared to M Tg and all WT.	*η*^2^ = 0.03 (Compared to M Tg)	
					*η*^2^ = 0.02 (Compared to F WT)	
✘	4, 6–6.5	3xTg-AD	M, F	No difference in CORT after restraint stress in M Tg and M WT (4 mo.)	*η*^2^ = 0.17	Nguyen et al. (2020)
✓				F Tg had higher CORT after restraint stress compared to F WT (4 mo.)	*η*^2^ = 0.26	
✓				F Tg had higher CORT 30 and 60 min. After restraint stress compared to WT F (6–6.5 mo.)	*η*^2^ = 0.25	
✓	4	3xTg-AD	M	Tg M had higher levels of dexamethasone non-suppression compared to WT M.	*η*^2^ = 0.07	Várkonyi et al. (2022)
✓	9	3xTg-AD	F	Tg F have higher CORT compared to Tg M.	*p* < 0.05	Clinton et al. (2007)
✓				Tg F have higher CORT compared to WT F.	*p* < 0.05	
✓	12	3xTg-AD	M	Tg M basal CORT is higher compared to WT M following social stressor.	*p* < 0.05 (2 h.)	Rothman et al. (2012)
✓				(2 h.; 6 h. timepoints)	*p* < 0.05 (6 h.)	
✓	19	3xTg-AD	M	Tg M basal CORT is higher compared to WT M.	*p* < 0.05	Muntsant and Giménez-Llort (2021)

### 4.3. 5-HT in AD models

The monoaminergic system in general, and the serotonergic system specifically, have been implicated in HPA axis activation, AD pathogenesis, and cognition. The serotonergic system is vital and heavily implicated in the experience of anxiety and depressive symptoms in animal and human models (Yohn et al., 2017). Additionally, risk factors such as early-life stress can alter 5-HT transmission. For instance, rats that experience early-life stress show significantly altered 5-HT transmission (Van Riel et al., 2004). Specifically, 5-HT_1A_ receptor density has been reduced in AD, which could account for some anxiety and depression symptoms experienced in AD patients (Chakraborty et al., 2019). In rodent models of AD, reports indicate that one of the areas severely impacted by Aβ and tau burden is the raphe nucleus, which may account for the disruption in 5-HT system signaling and receptor density. Restoring the effectiveness of this system could promote psychological well-being (e.g., reduced anxiety and/or depression) and possibly delay cognitive impairments in AD patients.

### 4.4. 5-HT trends and treatments in AD models

SSRIs are often used often in scientific literature due to their widespread availability and history of treating neuropsychiatric disorders. Research has examined the effects of SSRIs in Tg AD animals, with some studies reporting improved cognition and behavioral performance (Wang et al., 2014; Jin et al., 2017; Ma et al., 2017; Marwari and Dawe, 2018; Torrisi et al., 2019; Zhou et al., 2019). Specifically, several studies in AD mouse models (APP/PS1, Aβ-injected C57BL/6 mice) report efficacy of SSRIs (e.g., fluoxetine) in restoring cognition and cell density in the hippocampus (Cirrito et al., 2011; Ma et al., 2017; Caruso et al., 2021). Additional studies report that treatments with SSRIs or 5-HT receptor agonists increase brain derived neurotropic factor production and stimulate neurogenesis (Santarelli et al., 2003). Multiple studies report reduced Aβ deposition in APP/PSI mice and APP treated cells that received short-term and long-term treatment with SSRIs (Fisher et al., 2016; Reddy et al., 2021). Specifically, in 3xTg models, administration of risperidone attenuates anxiety-like behaviors and cognitive deficits in the OFT and MWT, respectively (Nelson et al., 2007; Torres-Lista et al., 2015). However, there are issues in both consistency and translational ability of SSRIs’ effectiveness in reducing AD pathogenesis and improving cognitive and behavioral deficits. In the APP/PS1 model, studies report no change in Aβ deposition, nor cognitive or behavioral measures (Severino et al., 2018; Sivasaravanaparan et al., 2022; see Mdawar et al., 2020; for a review; refer to Table 9). Although treatments with SSRIs varied in AD rodent models, given that treatment with SSRIs worked in a subsection of AD animal models, both in reducing AD pathological burden and reducing cognitive and behavioral disturbances, more targeted 5-HT treatment may be more effective. Further, with the lack of behavioral measure consistency in some AD animal models, it is possible that a more specific 5-HT treatment could produce more consistent and translational outcomes.

**Table 9:** Summary of 5-HT trends in AD models.

Difference between WT and Tg	Age (months)	Model	Sex	5-HT changes observed	Effect sizes/significance (*η*^2^, *d*, *p*)	Authors
✓	1	C57BL/6 mice (injected with Aβ oligomer solution)	M	Tg M cortical cells treated with Fluoxetine and Vortioxetine exhibited decreased cell death	*p* < 0.05	Caruso et al. (2021)
✘	2–2.5	C57BL/6 mice (injected with Aβ_1-42_)	M	Treatment with Vortioxetine or Fluoxetine rescued memory impairment in Tg M	*η*^2^ = 0.29	Torrisi et al. (2019)
✓	8	APP/PS1	M	Tg M treated with Fluoxetine had reduced mean escape latencies in the MWT compared to Tg conrols.	*p* < 0.01	Zhou et al. (2019)
✓				Tg M treated with Fluoxetine has reduced Aβ plaque burden compared to Tg controls.	*p* < 0.01	
✘	9	APP/PS1	M, F	No significant difference between Tg controls and Tg-Fluoxetine mice in MWT acquisition.	*η*^2^ = 0.60	Wang et al. (2014)
✘				Fluoxetine decreased escape latency of WT and Tg mice	*η*^2^ = 0.06	
✓	16–17	APP/PS1	M	Tg M treated with Fluoxetine had decreased escape latencies compared to Tg M controls.	*p* < 0.05	Ma et al. (2017)
✓				Tg M treated with Fluoxetine has more neurons in the DG compared to Tg M controls.	*p* < 0.05	
✘	18	APP/PS1	NR	Tg mice treated with Paroxetine did not show reduced Aβ or microgliosis compared to Tg mice controls.	*p* > 0.05	Sivasaravanaparan et al. (2022)

*η*^2^ calculated according to Lakens, 2013. NR signifies non-reported statistical results. * Signifies that the effect size is not trivial, but the data needed to determine the direction of the trend were not reported. The impact of effect sizes is subjective. However, the scientific community generally defines Cohen’s d effect sizes as: ‘small’ *d* = 0.2, ‘medium’ *d* = 0.5, and ‘large’ *d* = 0.8 (Paterson et al., 2016). While eta squared (*η*^2^) effect sizes are ‘small’ *η*^2^ = 0.01, ‘medium’ *η*^2^ = 0.06, and ‘large’ *η*^2^ = 0.14 (Kotrlik and Williams, 2003). The purpose of including effect sizes and significance measures in the above tables is to elucidate any consistent trends in behavioral testing in AD animal models.

## 5. Discussion

Overall, continuing improvements in AD animal models are crucial; more translational animal models will improve treatment efficacy, leading to better outcomes for AD patients. Additionally, early intervention is critical due to improved patient outcomes; however, diagnosing patients in the Preclinical/Prodromal Stage of AD presents challenges and concerns. Due to high sensitivity in more recent diagnostic measures, a false positive is more likely (Sperling et al., 2011). However, a focus on reducing psychological stress and anxiety, as well as improving HPA axis regulation, could still benefit individuals who receive a false positive (Plotsky et al., 1998). Animal models are important in pharmacotherapy development, but translational ability is perhaps the most important factor for researchers to consider. In this review, models of anxiety and locomotion were explored, including the OFT, LDT, EPM, and MWT. Of note, both 3xTg-AD and TgF344-AD had inconsistencies in behavioral and cognitive outcomes measured. In the 3xTg mouse model, the EPM was an unreliable measure of anxiety-like behavior, but the LDT findings remained consistent. Although many of these studies suggest that the EPM should not be used in this model, it is important to elucidate the reasons why the EPM is not reliable in these animals. Contrastingly, the EPM results were more consistent in the TgF344-AD rat model, suggesting that the EPM can be used for some AD animal models. Further, it is important to investigate mouse capabilities in wet tasks such as the MWT. Mice have been found to perform unreliably, anxiously, and slowly in the MWT; however, rats perform extraordinarily well in both wet and dry tasks, perhaps making them the preferred subjects for AD models (Whishaw and Tomie, 1996; Cohen et al., 2013). It is important to consider the age of the WT control rats as well, due to a steady decline in MWT performance (Lindner, 1997). Although this may explain some inconsistencies outlined in the present paper, the Tg animals should still demonstrate increased impairment compared to WT animals provided that control performance is not at the “floor.”

It is also important to consider potential differences in methodology, breeding, and housing. Although the same behavioral tests (EPM, MWT LDT, OFT) were conducted in each study, findings could be inconsistent due to variables such as housing, handling, breeding, and colony genetics. In the 3xTg-AD model, this may account for some variance observed in Tables 2–6 since the mice were all tested prior to 18 months, which is when Aβ, tau, microglia, and LTP deficits are seen in the more contemporary 3xTg-AD colonies (Javonillo et al., 2022). Although genetic drift has not yet been observed or reported in TgF344-AD rats, other factors, such as breeding and/or the amount of handling, could contribute to the varying behavioral results observed. Additionally, the behavioral testing apparatuses’, such as an EPM or open field, may differ between labs, which could also contribute to variance between studies. In order to increase standardization of research methods, scientists should include detailed methods sections allowing for more seamless replication of studies across laboratories.

Although inconsistencies were present in behavioral and cognitive measures in the AD models, both the 3xTg-AD and TgF344-AD animals consistently expressed robust AD pathology (Oddo et al., 2003; Cohen et al., 2013). Despite the extensive literature available on the 3xTg mouse model, the TgF344 rat model may be preferred in the future due to its more consistent behavioral findings and expression of neuroinflammation and neuronal loss, which remains lacking in mouse AD models. Additionally, mouse models rely on implantation of human tau while rat models rely on the naturally occurring rat tau gene (Cohen et al., 2013). Regardless of species chosen in the AD model, treatments with CRF or 5-HT ligands present a promising path forward. Due to the elevated cortisol and HPA axis activity consistently reported in animal AD models, there is opportunity to model stress and discover novel treatments (Ahmad et al., 2019). Targeting 5-HT could be an easy preventative measure to implement due to its widespread access and the high percentage of US adults having taken an antidepressant in their lifetime (Brody and Gu, 2020). Side effects of SSRIs can lead to noncompliance; however, more specific manipulations to both the 5-HT and CRF systems could lead to fewer side effects. Targeting CRF_1_ may improve AD outcomes in patients based on animal literature citing a reduction in AD pathology and studies showing anxiolytic responses to CRF_1_ antagonists. The specificity of medications, such as CRF_1_ antagonists, may limit side effects further while still improving clinical outcomes in AD patients.

## Author contributions

NR, NP, and BC produced original conceptualization of the current manuscript topic. NR performed literature searches, selected relevant papers, and wrote the manuscript. All figures made by NR using BioRender.com. DH provided table formatting and effect size calculation assistance. BC, DH, and NP were involved in critically reading and editing the manuscript. All authors contributed to the article and approved the submitted version.
